# Better Remuneration for Scientific Skill Recognized

**Published:** 1896-02

**Authors:** 


### EDITORIAL.

### BETTER REMUNERATION FOR SCIENTIFIC SKILL RECOGNIZED.

Recent losses of valuable services to the Department of Agriculture makes very potent the reference of Secretary Morton to the fact that there is a large number of employés who do only clerical work of a low grade, who are overpaid for such services, while, on the other hand, where scientific and technical skill is demanded, the compensation is inadequate, and not equal to that paid by commercial houses, corporations, and individuals, for like services. Hence it follows that ambition to attain a high place and worth are not stimulated or encouraged. Only too true, Mr. Secretary, but it has been ever thus, and every village and hamlet has its quota anxious to work in government positions, because of the greater pay and shorter time required than similar services command in the commercial centres of employment. Wipe it out, Mr. Secretary, for it is only an outgrowth of the pernicious spoils-system, and one of the grotesque pictures of American politics, where the scent of spoils makes party success stand higher than good government or the enforcement of righteous principles.

